# Gene Family Evolution across 12 *Drosophila* Genomes 

**DOI:** 10.1371/journal.pgen.0030197

**Published:** 2007-11-09

**Authors:** Matthew W Hahn, Mira V Han, Sang-Gook Han

**Affiliations:** 1 Department of Biology, Indiana University, Bloomington, Indiana, United States of America; 2 School of Informatics, Indiana University, Bloomington, Indiana, United States of America; University of Oxford, United Kingdom

## Abstract

Comparison of whole genomes has revealed large and frequent changes in the size of gene families. These changes occur because of high rates of both gene gain (via duplication) and loss (via deletion or pseudogenization), as well as the evolution of entirely new genes. Here we use the genomes of 12 fully sequenced *Drosophila* species to study the gain and loss of genes at unprecedented resolution. We find large numbers of both gains and losses, with over 40% of all gene families differing in size among the *Drosophila*. Approximately 17 genes are estimated to be duplicated and fixed in a genome every million years, a rate on par with that previously found in both yeast and mammals. We find many instances of extreme expansions or contractions in the size of gene families, including the expansion of several sex- and spermatogenesis-related families in D. melanogaster that also evolve under positive selection at the nucleotide level. Newly evolved gene families in our dataset are associated with a class of testes-expressed genes known to have evolved de novo in a number of cases. Gene family comparisons also allow us to identify a number of annotated D. melanogaster genes that are unlikely to encode functional proteins, as well as to identify dozens of previously unannotated D. melanogaster genes with conserved homologs in the other *Drosophila*. Taken together, our results demonstrate that the apparent stasis in total gene number among species has masked rapid turnover in individual gene gain and loss. It is likely that this genomic revolving door has played a large role in shaping the morphological, physiological, and metabolic differences among species.

## Author Summary

Though comparative genome sequencing has revealed vast similarities in the total number of genes contained within closely related species, this similarity hides enormous complexities in the identity and number of constituent proteins. Species can differ in their complement of genes through both gene duplication and loss. Here we investigated the gain and loss of genes from the genomes of 12 fully sequenced *Drosophila* (fruit flies). We find high rates of gain and loss in all species and estimate that approximately one new gene is gained or lost every 60,000 years. We also find several hundred cases of extremely rapid gene turnover, with dozens of genes gained or lost in only a few million years. The highest turnover in gene number occurs in genes involved in sex and reproduction. Taken together, our results demonstrate that the apparent stasis in total gene number among species has masked rapid turnover in individual gene gain and loss. It is likely that this evolutionary revolving door has played a large role in shaping the morphological, physiological, and metabolic differences among species.

## Introduction

A major goal of evolutionary genetics is to understand the molecular changes underlying phenotypic variation within and between species. The sequencing of whole genomes has made it possible to study not just individual mutations between orthologous sequences, but large-scale differences in gene complements between species. Such comparative genomic studies have found large disparities among organisms in the number of copies of genes involved in distinct cellular and developmental processes (e.g., [1,2]) and have even revealed the loss of entire gene families from individual lineages (e.g., [3,4]). Though these studies begin to offer some insight into the molecular basis for phenotypic evolution, the timescales considered are often too long to provide evidence for the role of any single change (but see, e.g., [5–8]). The sequencing of the genomes of 12 *Drosophila* species—whose most recent common ancestor (MRCA) lived only 60 million years ago [9]—offers the ability to study changes in the genomic complement of genes at an unprecedented resolution.

Changes in the number of genes and proteins devoted to specific biological processes may arise in a number of different ways. First, gene duplication along any lineage will increase the number of genes, resulting in gene families containing multiple copies that are partially or completely overlapping in function. These gene duplicates may subsequently diverge in function by taking on new roles or by dividing up old roles [10–12]. There are now numerous examples in *Drosophila* of individual gene families with duplicates differentiated in both protein sequences (e.g., [13–16]) and gene expression domains (e.g., [17]). A second reason for differences in gene complement among species is that genes may be lost along a lineage when disabling mutations in them are not selected against. Such gene losses can even be directly advantageous [18], consistent with the so-called “less is more” hypothesis of Olson and colleagues [19]. Finally, the de novo creation of genes through various processes (e.g., [20–22])—while certainly quite rare—may contribute to lineage-specific differences in the number and function of constituent proteins.

To provide a *Drosophila*-wide perspective on gene family evolution, we applied two different computational methods that estimate the rate and number of gene gains and losses. The first is a likelihood approach that estimates the average rate of gene gain and loss, the number of gains and losses on each branch of a phylogeny, and assigns *p-*values to large changes [23]. The second is the nonparametric gene tree/species tree reconciliation approach [24–27], which counts the number of gains and losses on each branch of the phylogeny without a specific probability model. While previous estimates of genome-wide rates of duplication in D. melanogaster [28,29] have offered a snapshot of one of the major mechanisms contributing to genome evolution, our analyses afford a wider view of this process. We show that genes have been gained and lost in all species at varying rates; that several hundred gene families exhibit significantly large expansions or contractions in number suggestive of adaptive natural selection; and that approximately equal numbers of gene families have either been lost completely in a species or are present only in a subset of the species considered here, information that can be used to improve the annotation of the D. melanogaster genome. Throughout the analyses we examine the effect that heterogeneity in both assembly and annotation quality among the 12 genomes can have on evolutionary inferences.

## Results/Discussion

### Gene Families in *Drosophila*

Using the predicted gene sets from all 12 *Drosophila* species, fuzzy reciprocal BLAST (FRB) was used to cluster genes into gene families on the basis of protein sequence similarity (Materials and Methods). All 188,868 genes in the dataset are assigned membership to a single family; the gene families are therefore nonoverlapping. Excluding lineage-specific families and likely annotation artifacts (see below), there are 11,434 gene families inferred to have been present in the *Drosophila* MRCA (“Analysis” in Table 1). The mean number of genes in each family is 12.97 (i.e., there is slightly more than one copy per species), with the largest family containing 144 copies across all 12 genomes. Although the term “gene family” often only refers to multiple, closely related paralogs within a species, we use the term here to denote groups of related genes that include both paralogs within the same species and orthologs and paralogs from other species. This broader definition makes it possible to study the evolution of gene families across species, as every sensu stricto gene family must have first appeared as a single-copy family [23].

**Table 1:**
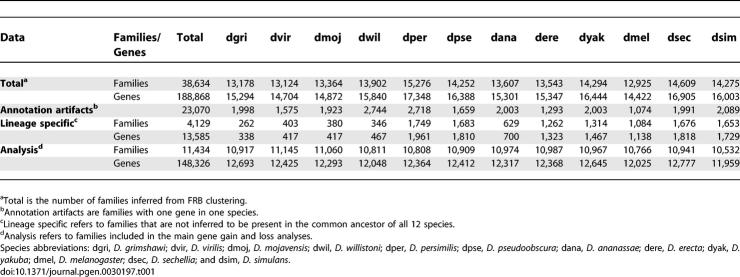
Number of Genes and Families in Each *Drosophila* Species

Of the 11,434 families, 4,693 (41.0%) have changed size in at least one species. There are no Gene Ontology (GO) terms that are over-represented among the families that have changed in size relative to the whole genome. The 4,693 families represent the minimum number that have undergone the gain or loss of genes, as equal numbers of gains and losses along a lineage will not result in a net change in family size. Different definitions of gene families may also affect results, as more stringent similarity thresholds make families smaller on average and less stringent thresholds make families larger [8]. To study the effect of changing gene family definitions, we reclustered the *Drosophila* genes by varying the BLAST similarity threshold used by an order of magnitude higher and lower (Materials and Methods). As expected, a more stringent similarity criterion caused there to be more, smaller families overall, but fewer families inferred to have been in the MRCA (8.0% fewer families), while a more lenient criterion caused there to be more families in the MRCA (9.8% more families). Changing the clustering thresholds also slightly changed the proportion of families changing in size in the expected directions—1.9% fewer changed when there were smaller families, while 2.1% more changed with larger families.

Any analysis of gene presence and absence must also consider the quality of the genomic data used to infer gene gains and losses [8]. There are two main sources of differences in data quality among the *Drosophila* genomes considered here: heterogeneity in gene prediction (“annotation”) and heterogeneity in genome coverage (“assembly”). We discuss the effect of each of these in turn.

The first *Drosophila* genome to be sequenced, D. melanogaster [30], is 99% complete at the sequence level and is in its fifth major annotation release after a number of years of manual curation [31]. For the purposes of the comparative analyses undertaken by the consortium analyzing the 12 *Drosophila* genomes [32,33], the most recent versions of the genome assembly and gene annotations are taken as the D. melanogaster gene complement (Berkeley *Drosophila* Genome Project release 5, http://www.fruitfly.org ). The ab initio gene prediction programs used to find genes in the other *Drosophila* species were not used as a basis for the final gene set from D. melanogaster. Likewise, similarity-based searches for finding genes in the other *Drosophila* species utilized already predicted genes from D. melanogaster, but not vice versa (but see [33] for an additional list of newly annotated D. melanogaster genes not included in release 5). The result of this heterogeneity in gene annotation is consistent with the known high false-positive rate of ab initio predictors: D. melanogaster is predicted to have the fewest genes of any genome by far (Table 1). Many more of the genes in the other 11 species are also found in gene families by themselves and are called annotation artifacts in our analyses (Table 1). In fact, there is a significant correlation between the total gene count from each genome and the number of single-gene, single-species families (*r* = 0.62, *p* = 0.033). Removing the thousands of genes without significant similarity to any others brings the predicted gene numbers among species much closer to one another. Importantly, the overprediction due to ab initio gene-finding software does not affect our main analyses as we eliminate such annotation artifacts from the dataset considered.

While ab initio gene prediction has a unidirectional effect on gene number (i.e., more genes), low-quality genome assemblies can lead to both the addition and subtraction of genes. Genes may be missing simply because there are large holes in the assembled genome, while genes can be added if allelic diversity within the sequenced strain is wrongly assembled as duplicated loci (e.g., [34]). The majority of *Drosophila* genomes were sequenced to greater than 8× coverage (i.e., the number of nucleotides sequenced is equal to eight times the total genome length), though the D. sechellia and D. persimilis genomes were only done to 4×, as their close relationships to high-coverage genomes was thought to mitigate the need for deeper sequencing. In addition, the D. simulans genome assembly is a “mosaic” assembly of low-coverage sequencing of six inbred lines of this species [35]. As might be expected from the lower quality sequence assemblies that result from lower sequence coverage, both D. sechellia and D. persimilis are predicted to have a high number of annotation artifacts (1,991 and 2,718 genes, respectively). D. sechellia, which is only ∼5 million years diverged from D. melanogaster, is initially predicted to have 2,483 more genes than this well-annotated genome; we do not believe that there is any evidence outside the ab initio gene prediction programs for this massive increase in proteomic complexity. Furthermore, many of the genes initially identified as pseudogenes in the D. sechellia and D. simulans genome have subsequently been found to be sequencing errors ([36]; C. Jones, personal correspondence). Because errors in both genome assembly and gene annotation will lead to errors in the number of inferred gains and losses, we have repeated many of the analyses that follow excluding D. sechellia and D. persimilis.

### Estimating Gene Gain and Loss via Maximum Likelihood

Our likelihood approach estimates the average rate of gene turnover across the *Drosophila*, λ, to be 0.0012 gains and losses/gene/million years; this is the rate at which the size of a gene family is expected to either expand or contract over time because of gene gain or loss (see Materials and Methods and [23]). Varying the definition of gene families resulted in a change in rate of only ∼2%. In comparison, Lynch and Conery [28] estimated the rate of gene gain in D. melanogaster via an independent method as 0.0023 duplications/gene/million years, an estimate consistent with the one presented here. Our rate is also similar to the rate of gene gain and loss estimated from both yeast (λ = 0.0020; [23]) and mammals (λ = 0.0016; [8]) using the same likelihood method. These data therefore suggest that there is a remarkably similar rate of gene duplication and loss across eukaryotes, suggesting common molecular mechanisms among species. The estimated rate of gene duplication and loss in *Drosophila* implies that within a single genome, there are approximately 17 new duplicates and 17 new losses fixed every million years (0.0012 gains and losses/gene/million years × 14,000 genes). A study of duplicate genes formed by retrotransposition in *Drosophila* found a much lower rate: only 0.51 new duplicates per million years [37]. These data appear to indicate that the rate of functional gene duplication via unequal crossing-over and transposition is higher than that via retrotransposition.

**Figure 1:**
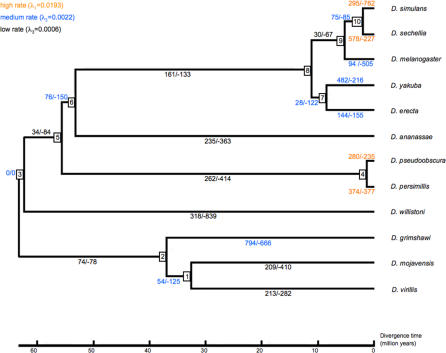
Gene Family Evolution in *Drosophila* On each branch of the tree the number of gene gains/losses is given. The colors of the numbers denote the estimated rate of gene gain and loss. Numbers in boxes are identifiers for internal branches of the phylogeny.

Estimating only the average rate of change across the phylogeny will mask any heterogeneity in evolutionary rates among species (e.g., [38]). We therefore attempted to estimate a fully parameterized model with 22 different values of λ, one for each branch of the tree, with an updated version of the program CAFE [39]. Though the likelihoods of estimated 22-parameter (22-p) models were consistently higher than that of the 1-p model, the results did not converge to a single global maximum (unpublished data). It is likely that the search space is simply too large to find such a maximum with 22 parameters. Instead, we created a 3-p model by assigning branches to one of three rate categories—fast (λ_ 1_), medium (λ_ 2_), and slow (λ_ 3_)—on the basis of the best branch-specific rate estimates from the 22-p model. This model always converged to a single maximum (λ_ 1_ = 0.0193, λ_ 2_ = 0.0022, and λ_ 3_ = 0.0006) and fit the data significantly better than the 1-p model (−2ΔL = 15,156; *p* < 1.0 × 10^−16^; df = 2; Figure 1). Although more parameter-rich models can be constructed, the distribution of rates estimated in the 22-p model suggested a natural division into three parameter classes; we also did not find that finer divisions offered any more biological insight than a 3-p model. The “fast” branches of the 3-p tree include the terminal lineages leading to D. simulans, D. sechellia, D. pseudoobscura, and D. persimilis. The “slow” branches include the terminal lineages leading to D. virilis, D. mojavensis, D. willistoni, and D. ananassae. Different definitions of gene families always significantly favored the 3-p model over the 1-p model.

It is important to note that the four rapidly evolving lineages are all either low-coverage genomes or are sister to low-coverage genomes (D. sechellia and D. persimilis); this is likely to contribute to the apparent rate increases. To ask whether the inclusion of these species has had a large effect on our inferences, we reestimated a 1-p model without D. sechellia and D. persimilis. As expected, the estimated average rate of gene gain and loss was lower without these two species, at λ = 0.0010 (compared to λ = 0.0012).

To ask whether the low-quality assemblies and annotations in these species have an effect on the number of gains and losses in closely related taxa, we compared two further models. In the first we estimated one rate for the D. melanogaster lineage (λ_ mel_) and one for all other branches (λ_ background_), including data from D. sechellia and D. persimilis. In the second model we estimated the same parameters but excluded the D. sechellia and D. persimilis data. This analysis reveals little difference in the estimated rate in D. melanogaster. Including the two questionable genomes gives λ_ mel_ = 0.0054 and λ_ background_ = 0.0011; excluding these two species gives λ_ mel_ = 0.0050 and λ_ background_ = 0.0010. These analyses demonstrate that the rate of gene turnover inferred in D. melanogaster is likely not an artifact of its relationship to D. sechellia, though the reduced dataset still includes the mosaic assembly of D. simulans. We therefore conclude that while poor annotation and assembly can have insidious effects on the inferred rate of gene gain and loss in affected genomes, these consequences should not reach far beyond the implicated lineages.

One further pattern revealed in the heterogeneous rates of gene gain and loss across lineages is the apparent relationship between branch length and rate. Though our previous analyses suggest that the high rates on the very short D. sechellia, D. simulans, D. persimilis, and D. pseudoobscura lineages are likely due to problems of annotation, many of the “medium” rate branches are also short in length (Figure 1). To ensure that the higher rates estimated on shorter branches of the tree are not due to a methodological artifact of our likelihood method, we simulated 1,000 datasets across the *Drosophila* tree under a 1-p model and then estimated rates of change under the same 3-p model as above (Materials and Methods). The average ratio of λ_1_/λ_3_ in these simulations was 1.00 and the maximum was 1.25, compared to the observed value of λ_1_/λ_3_ = 32.2. Also as expected if the likelihood ratio tests are χ^2^-distributed with 2 df, 5.7% of the simulated datasets had −2ΔL > 5.99 (i.e., *p* < 0.05). These simulations imply that the observed likelihood ratio (−2ΔL = 15,156) is highly significant (*p* << 0.001). Together, our results strongly suggest that the observed rate heterogeneity in the data is not due to a methodological problem.

Though the apparent negative correlation between rate of gene turnover and branch length is not due to an artifact, it is worthwhile to consider biological explanations for this relationship beyond the effects of genome annotation. Many of the shortest branches in the *Drosophila* phylogeny are also those closest to the tips of the tree. Because all comparative genomic studies—whether of nucleotide substitutions or gene gains and losses—use only a single genome from each species, estimates of divergence by necessity also include the polymorphisms present in the individual chosen for sequencing (even when this individual is highly inbred). If many segregating polymorphisms are slightly deleterious, then estimates of rates on tip branches may be higher than for deeper branches [40], though population sizes must be extremely large for this explanation to hold [41]. As studies of both humans (e.g., [42]) and *Drosophila* (J. J. Emerson and M. Cardoso-Moreira, personal correspondence) have uncovered a high number of polymorphic duplications and deletions of genes in natural populations, it is possible that these polymorphisms play a role in the higher rates of change seen in more recent lineages.

By estimating the maximum likelihood value for the size of gene families at internal nodes of the phylogenetic tree, we can infer the minimum number of gene gains and losses along each branch by comparing parent and daughter nodes ([8]). Doing this comparison for each branch of the *Drosophila* tree and summing across families allows us to estimate the total number of genes gained and lost along every lineage (Figure 1). Gains and losses of genes have occurred on all but one branch of the *Drosophila* tree (branch 3), and each terminal lineage leading to an extant species includes hundreds of gains and losses.

On the terminal lineage leading to D. melanogaster, we infer the gain of 94 genes and the loss of 505 genes in the ∼5 million years since the split with the simulans/sechellia clade. Running our analyses using alternative tree topologies [43] produced very similar results (unpublished data). The most common GO terms associated with gene families that have expanded in D. melanogaster are: proteolysis, defense response, cytoskeleton, extracellular transport, response to toxin, and trypsin activity. The most common GO terms associated with contracting gene families are regulation of transcription, protein binding, transcription factor activity, zinc ion binding, nucleus DNA binding, and mesoderm development. There are no significantly over-represented terms among these families.

The observed “revolving door” of gene gain and loss [8] has important implications for divergence among *Drosophila* species. For instance, even though the average synonymous site distance between D. simulans and D. melanogaster is 0.117 [35], D. melanogaster also has 856 genes that are not found in D. simulans (94 gains in D. melanogaster + 762 losses in D. simulans), and D. simulans has 800 genes not found in D. melanogaster (295 gains in D. simulans + 505 losses in D. melanogaster). This amounts to 5.9% divergence (856 + 800/2 × 14,000 genes) at the level of whole genes. These results imply that both changes in homologous nucleotides and the gain and loss of genetic material may be important in the differentiation of these two species (e.g., [44]).

### Estimating Gene Gain and Loss via Gene Tree/Species Tree Reconciliation

**Figure 2:**
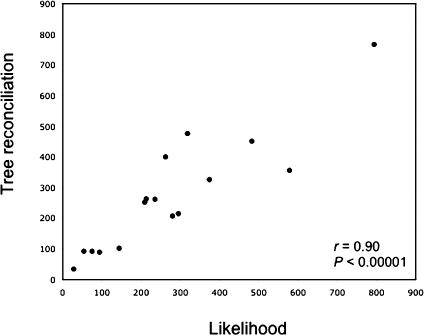
Correlation between the Number of Gene Gains on Informative Branches of the Phylogeny Inferred from the Likelihood Method and from the Tree Reconciliation Method

An alternative method for inferring the history of gene gain and loss among genomes is to reconcile the species tree with the gene tree of each family [24–27]. As this method does not assume a particular probability model for gains and losses, it is a valuable independent approach to estimating gene gains and losses. Tree reconciliation has frequently been used to infer gains and losses in individual families (e.g., [45]), but has been used less often to infer whole genome patterns of gene turnover (e.g., [38,46]). We built 11,390 gene trees from the 11,434 families using protein distances and the neighbor-joining algorithm [47]. We did not build trees for families with greater than 250 copies in total. We reconciled the 11,390 gene trees with the *Drosophila* species tree (as well as the two alternative species tree topologies) to map gene gains and losses to individual branches of the phylogeny (Figure S1). As a way of checking for consistency between the likelihood and gene/species tree approaches, we compared the number of inferred gene gains on informative branches from each (see Materials and Methods and [38]). The number of losses inferred by tree reconciliation methods can be highly biased because incorrect gene tree topologies will always add additional loss events towards the tips of the species tree [38], and therefore we do not use these estimates here. The correlation between the two methods was high (*r* = 0.90, *p* < 0.00001; Figure 2), indicating that our estimates of the number of gene duplications along each lineage are likely to be quite accurate. We inferred the gain of 89 genes in D. melanogaster since its split with simulans/sechellia using the tree reconciliation approach, compared to the estimate of 94 genes using the likelihood method.

The comparison between the tree reconciliation and likelihood methods also allows us to make some tentative conclusions regarding the frequency of gene conversion among *Drosophila* gene duplicates. Because gene conversion between duplicated genes will cause them to be highly similar, gene trees built from such genes will tend to show many more recent duplications. Even when there has been no change in the number of genes in a particular family, gene conversion will cause tree reconciliation methods to infer multiple, parallel duplications across lineages. This implies that rampant gene conversion will cause reconciliation methods to estimate many more duplications than our likelihood method, which is based only on the size of gene families. However, this is not seen (Figure 2): in fact, the ratio of genes estimated via reconciliation to that estimated via likelihood is 1.01, and more genes are estimated via reconciliation on only three of the 12 tip branches. Though these data certainly cannot rule out a role for gene conversion in individual families, they strongly suggest that it is at most a minor role genome-wide.

As a further check on the number of duplicates specific to D. melanogaster inferred from the 11,390 trees, we calculated synonymous site distances between all candidate pairs of duplicates in this species. If *d*_S_ = 0.117 is the average synonymous distance between D. melanogaster and D. simulans [35], then melanogaster-specific duplicates should be more similar than this. There are two explanations for why pairs of duplicates with greater divergence than expected (i.e., *d*_S_ > 0.117) can be inferred to be melanogaster specific using the tree reconciliation method. They may in fact be melanogaster specific but are evolving more rapidly at the nucleotide level than the average pair of orthologs; or the duplication event may pre-date the melanogaster-simulans split, but both D. simulans paralogs have been lost. As it is difficult to distinguish between these two possibilities, we have chosen to be conservative and to only count those pairs with *d*_S_ < 0.117. Of the 89 genes initially considered to be melanogaster-specific duplicates by tree reconciliation, 77 of them followed this rule. These should be considered a minimum estimate for the number of duplications unique to the D. melanogaster genome from these gene families.

### Accelerated Evolution of Gene Families

The likelihood approach to studying gene family evolution allows us to identify individual gene families that are evolving at rates of gain and loss significantly higher than the genome-wide average [23]. Such families can exhibit either larger-than-expected expansions or contractions, which may be confined to either a single lineage of the phylogeny or may reflect large changes across the tree. Of the 11,434 gene families inferred to have been present in the *Drosophila* MRCA, 342 exhibit significant expansions or contractions (*p* < 0.0001; Table S1). At this significance level, only slightly more than one family is expected by chance. We are especially interested in families with large, lineage-specific expansions, as it is likely that adaptive natural selection acts on lineage-specific traits through these changes [8,48,49].

Rapidly evolving families are associated with many biological processes, but the most common GO terms found among them are defense response, proteolysis, trypsin activity, protein binding, and zinc ion binding. Only one term—response to chemical stimulus (GO:0042221)—was significantly over-represented. Interestingly, many families in these categories have previously been identified as having large differences in copy number between both D. melanogaster and the mosquito, Anopheles gambiae [50], as well as between D. melanogaster and the nematode, Caenorhabditis elegans [2]. Our results demonstrate that there is significant variation in copy number even among closely related *Drosophila* species. It is also important to point out that genes involved in many of these processes (defense response, proteolysis, and trypsin activity) evolve rapidly at the protein level as well [32]. The parallel evolution of these proteins in sequence and copy number suggests that natural selection may act on multiple types of molecular changes to affect similar adaptive outcomes.

Of the 342 rapidly evolving families, we were able to identify 22 that showed large changes in copy number on the terminal branch leading to D. melanogaster (Table S2). Significant contractions occurred in 18 of the families and significant expansions in the remaining four (Dfam250, Dfam1703, Dfam2187, and Dfam6175). A total of four of the contracting families are made up of zinc-finger proteins, and all of the contractions in these four families result in complete loss of the family (i.e., there are no copies in the D. melanogaster genome). Family Dfam2548 has gone from five copies to one copy; the one remaining gene in D. melanogaster is longitudinals lacking (*lola*) and is involved in axon growth and guidance [51]. Another family to show a significant contraction (Dfam3206) was reduced from four copies to one copy (*pipe*) in D. melanogaster and is reported to be involved in embryonic pattern formation. There are many additional families that have been lost from D. melanogaster (see Loss of Entire Gene Families, below), but none show such dramatic reductions in number in the last five million years.

The four families with significant expansions have varying biological functions, though all may be involved in reproduction: one contains analogs of the protein kinase CK2 complex (Dfam2187), one is the Sdic (sperm-specific dynein intermediate chain) gene family (Dfam6175), and two are proteolysis/trypsin families (Dfam250 and Dfam1703). (The Dfam database, containing descriptions of the families, alignments, gene trees, and links to FlyBase can be found at http://www.bio.indiana.edu/∼hahnlab/Databases.html.) The family annotated as protein kinases has expanded in number from four to 14 in D. melanogaster. This family contains the gene Stellate (*Ste*), which is involved in male fertility and meiotic drive [52,53] and is arranged in tandem repeats on the X chromosome in D. melanogaster [54]. It was previously thought to have been absent from other species in the melanogaster group of *Drosophila* [54], though we find homologs in all 12 *Drosophila* genomes considered here. New gene duplicates in the Sdic gene family were previously reported to have been fixed by adaptive natural selection [55,56]. This family is made up largely of duplicated genes that originated as a chimeric fusion between the *Cdic* and *AnnX* genes, and that are newly expressed in the testes of male D. melanogaster [55,57]. Here we find that this family has expanded from two copies (including the progenitor *Cdic* genes) to five copies in D. melanogaster.

The two other families that show rapid expansions in D. melanogaster also have reproduction-related functions. Both families of proteolysis/trypsin genes have gained two gene duplicates; Dfam250 has gone from five to seven copies and Dfam1703 from seven to nine copies. Dfam250 shows some evidence for positive selection on the melanogaster-specific protein sequences (*p* = 0.05), while Dfam1703 does not. As discussed earlier, proteins with trypsin activity are often found to evolve via adaptive natural selection; it is likely that this high rate of sequence evolution is due to their role in male–female sexual antagonism [58]. Consistent with our observation of rapid evolution in this family in both copy number and protein sequence, we found another family containing trypsin genes that had a significant expansion along lineages leading to D. melanogaster. Dfam239 experienced an expansion from 20 to 28 copies along the branch leading to the melanogaster group (branch 6; Figure 1) and a second large expansion from 28 to 46 on the branch leading to the melanogaster subgroup (branch 8; there are 46 members of this family found in the D. melanogaster genome). We also found strong evidence for positive selection on the protein sequences of this family (p < 0.001).

The coincidence of positive selection on protein sequences with expansion of gene number in the above families led us to investigate this relationship further. We analyzed all 49 families that contained D. melanogaster-specific duplications for evidence of positive selection (these families contain the 77 new gene duplicates). Again comparing nonsynonymous to synonymous distances among the paralogs, we found that models including positively selected sites (M2a in PAML) were significantly favored over models without positive selection (M1a) in ten families (20.4%; *p* < 0.05, df = 2). Of these, six were significant after Bonferroni correction (*p* < 0.001). Friedman and Hughes [59] found a similarly high fraction of positively selected duplicates in a comparison of human and mouse, but interpreted their result as a bias in the likelihood method. They further proposed that this bias becomes worse as divergence times grow between sequences. As a comparison, therefore, we examined the frequency of positive selection found among single-copy orthologs in *Drosophila* using the same methods [32]. As expected, only 309 (3.6%) of 8,510 sets of orthologs showed evidence for positive selection. As the orthologs have much deeper divergence times than the melanogaster-specific duplicates, we believe that our results uncover a real biological pattern and are not the result of biased methods. However, despite the fact that we have found little evidence for gene conversion among duplicates, if present it may cause false rejection of the null hypothesis [60]. The high fraction of positively selected duplicates observed in D. melanogaster is consistent with genome-wide comparisons in rhesus macaque [49] and a number of individual studies from *Drosophila* (e.g., [15,21,61]). Whether this selection acts initially to fix duplicates or acts after fixation on unconstrained protein sequences is unknown; either way, it suggests that adaptive protein evolution is a frequent feature of duplicate gene evolution [10].

### Loss of Entire Gene Families

**Figure 3:**
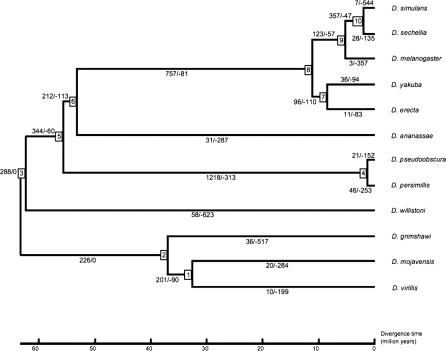
Lineage-Specific and Extinct Gene Families On each branch the number of lineage-specific families/extinct families are given. Numbers in boxes are identifiers for internal branches of the phylogeny.

Gene loss occurs in almost every family that changes in size. Sometimes this results in complete loss of a family: 2,220 of the 11,434 families inferred to have been present in the *Drosophila* MRCA have had such an extinction event along at least one lineage. The remaining 9,214 families are present in all 12 *Drosophila* genomes and should be considered the “core” proteome of these species. In total, we infer a minimum of 4,399 contractions that result in the complete loss of a family (multiple extinctions can occur within a single family along distinct lineages), occurring on every branch of the phylogeny (Figure 3). This number represents a rate of 12 extinctions per million years (=4,399 extinctions/367 million years total in the tree). Varying the similarity threshold used to define gene families did affect the number of extinctions, but order-of-magnitude changes in this threshold only changed the number of extinctions 6%–7% in either direction.

The D. melanogaster genome has lost 668 entire gene families that are present at the root of the *Drosophila* tree; 357 of these families have been lost from only the D. melanogaster genome (Figure 3). Families that are lost from the D. melanogaster genome have many of the same functions as those that are lost from other species. The most common GO categories among extinctions across the *Drosophila* include zinc ion binding, proteolysis, protein binding, and transcription factor activity. None of these are significantly over-represented.

The loss of entire gene families has been previously observed in many taxa (e.g., [4,5,8]). Results from these studies indicate that while the apparent loss of whole gene families can result from the true loss of all functional genes, there are multiple alternative explanations, including being an artifact of the threshold used for clustering [4,8], or missed annotations of genes present in completed genomes. For the families that appear to be extinct in D. melanogaster, we attempted to distinguish among true extinctions, clustering artifacts, and possible missed annotations.

Of the 357 families that appear to have gone extinct along the D. melanogaster branch, 292 have a homologous gene present in D. simulans. We used TBLASTN to search the D. melanogaster genome for sequences with high similarity to these D. simulans genes, and further asked whether matching sequences were syntenic with the D. simulans genes. If matching D. melanogaster sequences were not previously annotated as genes, we used GeneWise [62] to predict gene models (see Figure S2 for a summary of results). Though there are many ambiguous cases, we found four extinctions (1.4% of all extinctions) that appear to be artifacts of the clustering algorithm: previously predicted D. melanogaster genes that were syntenic with the D. simulans query sequence and that were members of families with more D. melanogaster than D. simulans genes (such that additional extinctions did not have to be introduced by shifting genes between families). One of these D. melanogaster genes (CG6908) is evolving at ∼3.5 times the average nonsynonymous rate and may therefore represent an “extinction” of function without loss of a physical gene. Of the 292 extinctions, we were further able to predict 98 previously unannotated genes in D. melanogaster that had both good matches to predicted genes from D. simulans as well expressed sequence tag (EST) or other expression evidence (Table S3). Of these, 62 match novel gene predictions using other methods [33], and 17 match third-party annotations in National Center for Biotechnology Information (NCBI) that were not included in FlyBase (Figure S2; Table S3) [63]. The majority of previously unidentified genes reside in the 5′ UTRs of annotated genes and are therefore likely to be missed by ab initio gene prediction programs. Our results suggest that while there may be many true losses of entire gene families, taking advantage of comparative genomic data may help to uncover many previously unannotated genes. And though these data indicate that we have overestimated the number of extinctions because of missed annotations, this problem may be largely confined to the D. melanogaster genome, where ab initio gene predictors were not used.

### Lineage-Specific Gene Families

When the MRCA of the *Drosophila* is not inferred to have contained any members in a gene family, we conclude that the family evolved subsequent to the MRCA of the species considered. Only species descended from the ancestor in which the family evolved would then have any gene copies. Such lineage-specific families (also called “orphans” [64–66]) may arise for a number of reasons: (1) the de novo evolution of new genes [67]; (2) rapid protein evolution in previously existing genes so that they are no longer identified as being part of a pre-existing family [8,65,66]; (3) artifacts of the clustering process [8,64]; (4) horizontal gene transfer [68]; (5) extinctions on a majority of lineages considered [8]; or (6) incorrect annotations of sequenced genomes [65].

We considered families to be lineage specific if they were not found in at least one species of both the *Sophophora* and *Drosophila* subgenera and were also present in at least two copies (see Materials and Methods). These criteria result in 4,129 families that we considered to be lineage specific, implying the creation of 11 new gene families per million years (=4,129 lineage-specific families/367 million years total in the tree). These families have evolved on every branch of the tree and in every species (“Lineage Specific” in Table 1 and Figure 3). As expected [8], varying the similarity threshold used to define gene families also changed the apparent number of lineage-specific families: a more stringent threshold led to 1.4% more lineage-specific families, while a less stringent threshold led to 1.9% fewer.

Of the 493 lineage-specific families in the subgenus *Drosophila*, 226 are found in all three species. Of the 3,636 lineage-specific families in the subgenus *Sophophora*, 288 are found in all nine species. The large difference in the number of families unique to each subgenus is likely due to the unequal sampling of species: extinctions on the relatively longer branch leading to the subgenus *Drosophila* species, for instance, will result in many families that appear to be specific to the *Sophophora*. Similarly, the way in which we define lineage-specific families relative to annotation artifacts—that they must be present in multiple copies—likely leads to a large number of lineage-specific families apparently originating on the lineages leading to D. pseudoobscura/D. persimilis and D. simulans/D. sechellia: close relationships between these sister species mean that even spurious gene predictions will have highly similar homologs.

We found three families with multiple gene copies that are unique to D. melanogaster (Dfam12771, Dfam14517, and Dfam15564). The largest of these families has five members (Dfam12771), but no known annotation in FlyBase or via a search of the Pfam database [69]. Pfam annotations of the other D. melanogaster-specific families reveal proteins involved in puparial adhesion and exocytosis. Over-represented GO terms associated with lineage-specific families in all species include trypsin activity, proteolysis, and postmating behavior (Figure S3; Table 2). These terms are noteworthy, as previous work has uncovered evidence for the evolution of truly de novo proteins with the same functions (e.g., [22]), though they are also a rapidly evolving group of proteins at the nucleotide level. Many of these de novo genes are expressed in the accessory glands of male *Drosophila* and are likely to have arisen from previously noncoding DNA [22]. Supporting this result, we find that our lineage-specific families contain proteins that are on average 50% shorter than the majority of *Drosophila* proteins (277 versus 551 amino acids; *p* = 2.6 × 10^−59^).

**Table 2:**
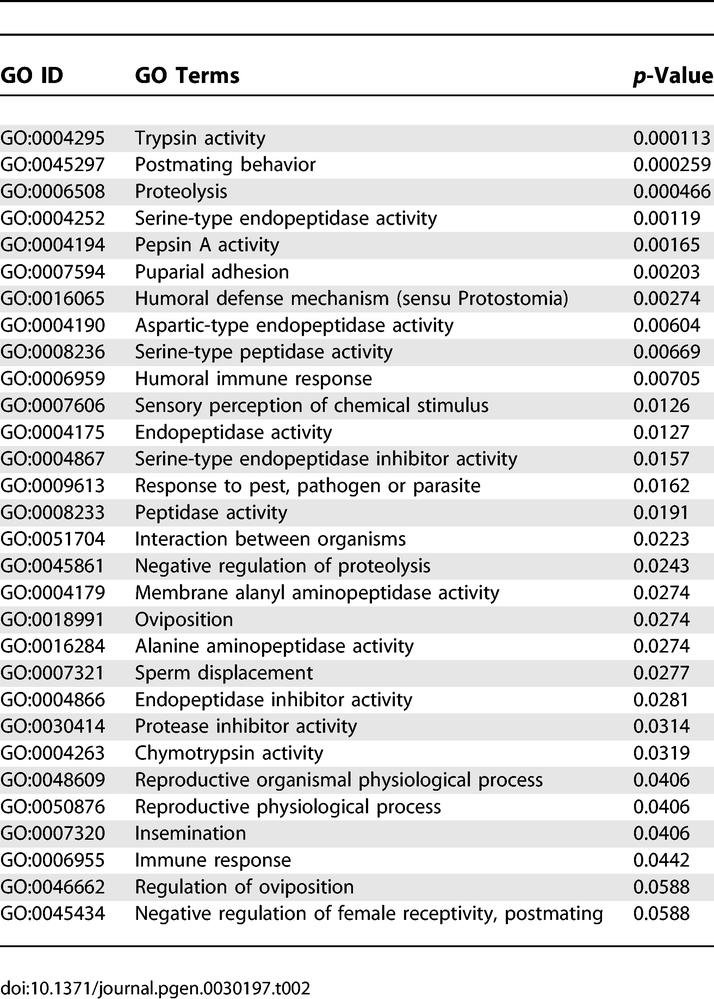
Over-represented GO Terms among Lineage-Specific Families

As noted above, previous work has found that some lineage-specific D. melanogaster genes appear to be incorrect annotations [65]. As the sequencing of multiple *Drosophila* genomes affords a much deeper comparative genomic dataset with which to address this question, we attempted to identify additional gene models from the D. melanogaster genome that have little evolutionary or functional support (see also [33]). We concentrated on genes found within single-gene, single-species families (“annotation artifacts”). Of the 1,074 genes (families) we previously called annotation artifacts in D. melanogaster, 716 were found to be RNA genes upon closer inspection. Of the 358 remaining genes, 94 had no EST support and no tBLASTX match in the D. simulans genome (Figure S4; Table S4). Many of these genes are quite short (average length of 319 amino acids), and are highly likely to be incorrectly annotated D. melanogaster genes. A total of 34 of these genes were also marked as bad annotations using other methods [33]. Finally, we found 15 cases where the D. melanogaster genes that we called annotation artifacts were: syntenic with a similar D. simulans gene; had EST matches in GenBank; had *d*_S_ < 0.20 to the matching D. simulans gene; and where the family containing the D. simulans homolog had more copies in D. simulans than D. melanogaster (suggesting that the “annotation artifact” might explain an apparent loss in D. melanogaster if included in this family). These genes have an average *d*_N_ = 0.041, compared to the average across all genes between these two species of *d*_N_ = 0.016 [35], and four have *d*_N_/*d*_S_ > 1. Though we have called these genes annotation artifacts, it appears more likely that they are simply extremely rapidly evolving genes.

### Conclusions

By studying the gain and loss of genes, we hope to better understand the forces that shape morphological, physiological, and metabolic differences among species. We have shown here that even among 12 closely related *Drosophila*, there have been a large number of gene gains and losses along each lineage, in proteins involved in a wide range of biological functions. There has also been the gain and loss of whole gene families, at approximately equal rates across the *Drosophila*. In the past 5 million years of D. melanogaster evolution, there has been the gain of at least 94 duplicated genes, some of these likely evolving by adaptive natural selection. In addition to garnering novel insights into genome evolution, studies of the gene complements of multiple *Drosophila* species can help to annotate the D. melanogaster genome. As demonstrated here, such analyses can improve the D. melanogaster annotation by either adding or removing genes from this genome. Though comparative genome sequencing has revealed vast similarities in the total number of genes among taxa, this similarity hides enormous complexities in the identity and number of constituent proteins.

## Materials and Methods

### Data.

Gene models across all 12 species are taken from the consensus set defined by the *Drosophila* Genome Sequencing and Analysis Consortium [32,33]. Gene families were assembled by a modified reciprocal BLAST method (FRB, [32]). Briefly, FRB proceeds by first performing all-by-all comparisons between the 12 genomes using BLASTP. Rather than taking only the top hit as the putative ortholog—as is done in most reciprocal BLAST methods—FRB considers proteins to be in the same “rank” if the absolute difference in successive BLAST E-values is less than two orders of magnitude (i.e., a difference in score of 100). This E-value threshold was changed when the data were reclustered to either a difference in E-values of 10 or a difference of 1,000. Genes in the same rank are potentially homologous, and the clustering step of FRB traverses the graph of pairwise relationships to find the maximally connected clusters that are disjoint from one another while discarding nonreciprocal relationships. These clusters include both orthologs and paralogs and are the gene families used in our analyses (description of FRB courtesy of V. Iyer).

In total this method identified 50,042 gene families in all 12 species, including 223,963 genes. After filtering out gene models predicted to be derived from transposable elements, the total numbers were reduced to 38,634 families containing 188,868 genes. We determined whether families were present in the MRCA, and if not, on which branch the family had originated. A family was defined as being present in the MRCA (with at least one gene copy), if it was found in at least one species of both the *Drosophila* (D. virilis, D. mojavensis, and *D. grimshawi*) and *Sophophora* (*D. willistoni, D. persimilis, D. pseudobscura, D. ananassae, D. erecta, D. yakuba, D. melanogaster, D. sechellia,* and *D. simulans*) subgenera. The branch on which families originated was determined by parsimony rules: if leaf branches share a family, the MRCA of those branches is regarded as the point of origin of the family. These are the same criteria by which losses of families were mapped onto the tree.

Using these rules, we found 23,070 families that consisted of a single gene and that appeared to have evolved on a terminal lineage (i.e., they are found in only a single species). These single-gene families were regarded as artifacts of the annotation process, and were removed from further analysis. We also found 4,129 families that arose after the split between the main two subgenera, but that were either found in multiple species or had multiple copies in one species. Since our likelihood analysis assumes that there is at least one ancestral gene in the MRCA (see below), we separated these families from the likelihood analysis. This left 11,435 families with at least two genes across the both subgenera. Close examination of the data revealed one family (Dfam8) predicted to be made up of >85% transposable elements. As it seems likely that the remaining ∼15% of gene in this family are also transposable elements, this family was removed from all downstream analyses, leaving 11,434 families for the final dataset used in the likelihood analysis.

### Likelihood analysis of gene gain and loss.

To estimate the average gene gain/loss rate and to identify gene families that have undergone significant size changes, we applied the probabilistic framework developed by Hahn et al. [23]. By using a stochastic birth and death model for the gene gain and loss across species and a probabilistic graphical model for the dependence relationship between branches of the phylogeny, this framework can infer the rate and direction of the change in gene family size. Assuming that all genes have equal probability λ of gain (birth) and loss (death), the conditional probability of going from an initial number of genes *X*_0_ = *s* to size *c* during time *t*, is given as,

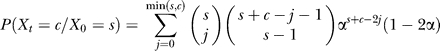
where, 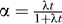 . Since *X*_0_ = 0 will result in a probability of zero for birth and death, we restrict our analysis to families in which *X*_0_ > 0. That means we exclude lineage-specific families from our likelihood analysis. A total of 11,434 families including 148,326 genes were analyzed. The phylogeny for the analysis was based on the tree found in [32].

The rate of gene gain and loss, λ, was estimated by an expectation-maximization algorithm that maximizes the sum of the log-likelihoods of each family. The likelihoods we want to maximize are the conditional likelihood of the observed family sizes given the root size. The ancestral family sizes at internal nodes are computed by averaging over all possible assignments during this maximization. For further details see Hahn et al. [23] and De Bie et al. [39]. We estimated three different models with varying numbers of parameters. A model with one global λ gave us a consistent result, while a model with 22 λ-parameters (one for each branch of the phylogeny) failed to converge to a single, consistent global maximum. On the basis of the best results for the 22-p model, we categorized branches into three rate categories: fast (>0.001), medium (0.001–0.0001), and slow (<0.0001).

To test for biases in parameter estimation, we used the estimated rate for the 1-p model (λ = 0.0012) to simulate data over the *Drosophila* phylogeny for each of the 11,434 gene families. Each of 1,000 simulations starts by setting the root sizes for all 11,434 families equal to the maximum likelihood size estimated from the dataset, and then evolving these families over the tree according the birth–death probability model described above. For each of the 1,000 simulated datasets we then estimate λ-values under both the 1-p and 3-p models. As the data were generated under a 1-p model, these simulations act as a null hypothesis against which results from the 3-p model can be compared.

To calculate the number of gene gains and losses on each branch of the tree, we compared the sizes of all parent–daughter node pairs (using the maximum likelihood ancestral gene family sizes). The difference in size between these two values was inferred to be the number of genes gained or lost: larger daughter sizes imply gene gains, while smaller daughter sizes imply gene losses. These numbers are minimum estimates, as gains and losses in the same family will result in fewer observable events. Total gains and losses were summed across all 11,434 families on all lineages.

Our likelihood approach also allows us to set up a null hypothesis against which we can compare the rate of evolution of individual gene families. Using the maximum likelihood parameters of the 3-p model, we ran Monte Carlo simulations to test for significant rate accelerations in all 11,434 families [23]. Using *p* < 0.0001, we expect there to be approximately one significant result by chance; the observation of 342 families with lower *p*-values implies a false discovery rate of 0.003%. To identify the branch of the *Drosophila* tree with the most unlikely amount of change for these 342 families, we calculated the exact *p*-values for transitions over every branch (the “Viterbi” method in [39]). We called individual branches significant at *p* < 0.005.

### Reconciling gene trees and species trees.

Alignments among proteins in each of the gene families were generated by MUSCLE [70]. A neighbor-joining tree was built for each family on the basis of the alignment and JTT protein distances using PHYLIP [71]. We were only able to construct gene trees for 11,390 of the 11,434 families (PHYLIP could not handle trees with more than ∼250 genes). Using the rooted species tree, we compared each gene tree with the species tree to map each node in the gene tree as either a speciation or a duplication event. With this information we can bound the date of each gene duplication to the resolution of each speciation event. The reconciliation of gene tree and species tree was done using the software NOTUNG [27] with 100% bootstrap cutoffs to collapse poorly supported topologies. By inferring the placement of duplications, we were able to estimate the number of gains on each branch of the species tree. Nodes with three or more descendant lineages are prone to overestimate the number of duplications on the branches ancestral to them [38]; we therefore excluded branches 2, 3, 5, 6, 8, and 9 from comparisons between the likelihood and tree reconciliation methods.

### Positive selection on nucleotide sequences.

We asked whether there was evidence for positive selection on the nucleotide sequences of *D. melanogaster*-specific duplicates using the ratio of nonsynonymous (*d*_N_) to synonymous (*d*_S_) substitutions per site. If *d*_N_/*d*_S_ > 1, then adaptive natural selection must be acting to fix nonsynonymous mutations. We compared the likelihood of models with no positive selection (M1a) to the likelihood of models with positive selection (M2a) in the program PAML [72]. The M1a/M2a comparison was used rather than more complex branch-site models so that the same test could be used on all D. melanogaster-specific duplicates: M1a/M2a does not require an outgroup to detect positive selection along the *melanogaster* lineage. The likelihood ratio test conservatively assumes 2 df because of boundary effects in parameter estimation [73].

### Annotation of gene families.

The basic annotations for each gene family were based on the FlyBase GO term database (FlyBase 4.3, http://flybase.bio.indiana.edu/). We searched this database using the D. melanogaster proteins. The most common GO terms in cellular component/function/process were identified, and a consensus set of terms was used if genes in the same family had different GO terms associated with them. If no annotation was retrieved for any of the genes in a family, we searched Pfam for matching protein domains. In total we were able to annotate 9,752 of the families, 7,460 via FlyBase and 2,292 via Pfam. The program GOstat [74] was used to find over-represented GO terms at each level in the GO hierarchy.

## Supporting Information

Figure S1 Gene Gain and Loss Using Tree Reconciliation Methods On each branch of the tree the number of gene gains/losses inferred by gene tree/species tree reconciliation is given. The number of gene losses using this method is highly biased [38].
(59 KB TIF)

Figure S2 Extinctions in D. melanogaster
The Venn diagram summarizes the results of searching for 292 extinct genes in D. melanogaster using D. simulans homologs. Genes predicted to be pseudogenes in each category are not shown. D.mel, D. melanogaster; D.sim, D. simulans; nr db, NCBI nonredundant database.
(90 KB TIF)

Figure S3 GO Hierarchy for Significant Terms GO terms significantly over-represented among lineage-specific families are highlighted in yellow.
(6.1 MB TIF)

Figure S4 Annotation Artifacts in D. melanogaster
The Venn diagram summarizes the results of searching for the 1,074 genes in D. melanogaster that were in families by themselves against the D. simulans genome. D.mel, D. melanogaster; D.sim, *D. simulans.*
(66 KB TIF)

Table S1 Rapidly Evolving Gene Families in *Drosophila*
The tree-wide *p*-values are given, as well as the individual *p-*values for changes along each branch of the tree, the inferred size of each family at bottom of each branch, and the inferred amount of change on each branch.
(271 KB XLS)

Table S2 Rapidly Evolving Gene Families in D. melanogaster
The current size of the families and the inferred number of changes since the split from the *simulans*/*sechellia* ancestor are given.
(23 KB XLS)

Table S3 Newly Predicted Genes in D. melanogaster
Genes overlapping with new predictions from Stark et al. [33] are listed with their CONGO IDs, while genes overlapping with third-party annotations from Hild et al. [63] are labeled “TPA.” NCBI identifiers for the EST matches to predicted genes, GeneWise prediction scores, and the D. simulans putative homolog IDs are also given.
(32 KB XLS)

Table S4 Genes from D. melanogaster Predicted to Be Incorrect Annotations Genes overlapping with predictions of incorrect annotations from Stark et al. [33] are listed with their CG number.
(25 KB XLS)

### Accession Numbers

The FlyBase (http://flybase.bio.indiana.edu/) accession number for *CG6908* is FBgn0037936.

## Abbreviations

EST: expressed sequence tag
FRB: fuzzy reciprocal BLAST
GO: Gene Ontology
MRCA: most recent common ancestor
-p: -parameter
